# The Benefits of an In-Office Arthroscopy in the Diagnosis of Unresolved Knee Pain

**DOI:** 10.1155/2018/6125676

**Published:** 2018-01-21

**Authors:** Garrett L. Chapman, Nirav H. Amin

**Affiliations:** ^1^Andrews Research & Education Foundation, Gulf Breeze, FL, USA; ^2^Loma Linda University Medical Center, Loma Linda, CA, USA

## Abstract

We report a patient who developed persistent knee pain with mechanical symptoms after an uncomplicated patellofemoral arthroplasty. The etiology of his knee pain remained inconclusive following magnetic resonance imaging due to metallic artifact image distortion. With the use of an in-office needle arthroscopy, an immediate and definitive diagnosis was obtained, preventing an unnecessary surgery for a diagnostic arthroscopy. We discovered a lateral meniscus tear, an anterior cruciate ligament tear, and a medial femoral condyle chondral defect for which the patient underwent arthroscopic partial meniscectomy, ligament reconstruction, and osteochondral allograft transplantation, with resolution of his knee pain.

## 1. Introduction

Knee pain is among the most common issues that orthopaedic surgeons diagnose and treat. Advanced imaging, including magnetic resonance imaging (MRI), is frequently obtained to aid in the diagnosis. Despite the superior soft tissue resolution of MRI, it is not infallible. This is particularly evident when orthopaedic implants are present, as metal artifacts can cause significant image distortion. In these situations, it is not uncommon for patients to be taken to the operating room for a diagnostic arthroscopy to assess the pathology for potential surgical planning. However, an alternative option exists which allows the surgeon to visualize the compartments of the knee in an in-office setting. This solution avoids the risk of anesthesia, delivers immediate answers to the patient's symptoms, and provides cost savings to the patient, hospital, and insurance company. We present a patient with persistent right knee pain in the setting of a previous patellofemoral arthroplasty. The patient was informed that data concerning his case would be submitted for publication, and consent was obtained.

## 2. Case Report

The patient, a 38-year-old active-duty Marine, presented to our clinic with a history of persistent right knee pain that occurred while going up and down stairs and was most prominent along the anterior knee. His medical history was significant for a diagnostic arthroscopy done elsewhere for a “clean up” of the right knee, which provided minimal pain relief. The operative report and images demonstrated Outerbridge grade IV changes involving the lateral patellar facet, grade III and IV changes along the medial patellar facet, and grade IV changes along the entire trochlea down to the intercondylar notch. Magnetic resonance imaging (MRI) of his right knee was obtained, which demonstrated ongoing patellofemoral osteoarthritis with a kissing lesion involving the trochlea and lateral facet of the patella with subchondral cyst formation. No evidence of new pathology was present elsewhere in the knee. After exhausting all conservative treatment options, he underwent an uncomplicated patellofemoral arthroplasty for isolated right knee patellofemoral degenerative joint disease.

Four months after surgery, the patient had regained full range of motion (ROM) of his right knee and returned to his previous activity level, including running and playing basketball. Furthermore, he was able to pass the required endurance tests for military active-duty reinstatement.

**Figure 1:**
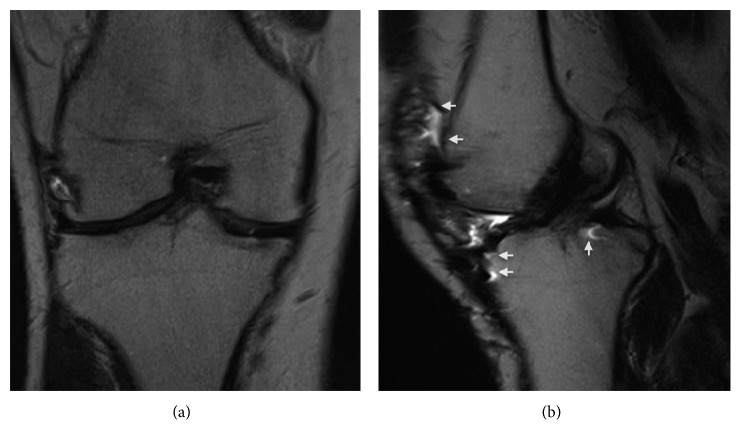
Coronal (a) and sagittal (b) T2-weighted metal reduction magnetic resonance images of the right knee, demonstrating an apparently intact ACL. Note the presence of metallic artifacts (white arrows) throughout the anterior and posterior aspect of the knee on the sagittal image.

**Figure 2:**
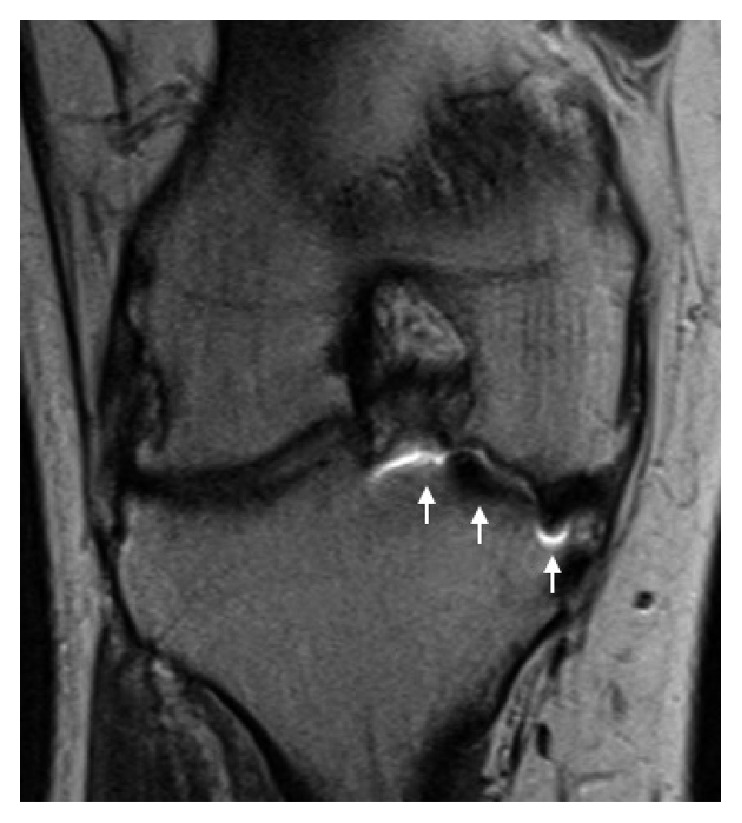
Coronal proton density with metal reduction MRI of the right knee demonstrates significant image distortion (white arrows) from metallic artifacts, obscuring accurate evaluation of the medial femoral condyle articular surface.

**Figure 3:**
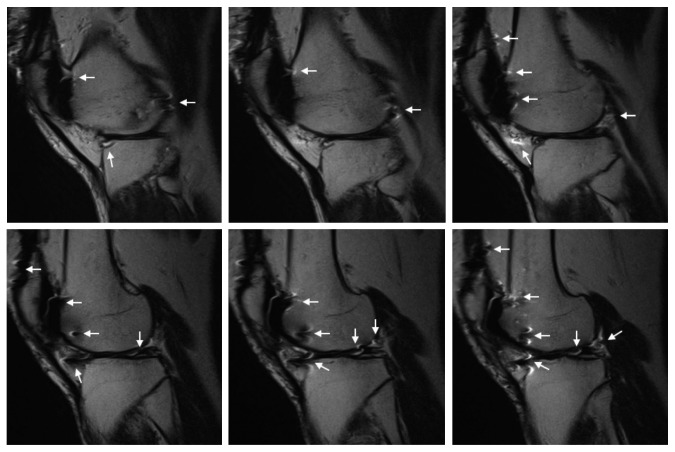
Sequential sagittal T2-weighted metal reduction MR images of the right knee lateral compartment. The presence of metal artifacts (white arrows) obscures accurate evaluation of the lateral meniscus.

Approximately ten months after surgery, he returned with moderate, sharp right knee pain, which he attributed to prolonged physical activity and playing sports. His knee pain was predominantly over the lateral joint line and was associated with catching and clicking. Clinical examination was significant for tenderness over the lateral joint line with positive Thessaly and McMurray tests. A mild effusion was present, and the ROM of the right knee was 0–125°. A stability examination revealed moderate (2B) laxity with Lachman and anterior drawer tests. No varus or valgus instability was present, and results of the dial and reverse pivot shift tests were normal. Based on his clinical examination and concern for lateral meniscus and anterior cruciate ligament (ACL) pathology, a metal reduction MRI was acquired. The images revealed an intact patellofemoral arthroplasty; however, evaluation of the ACL and menisci was nondiagnostic due to metallic susceptibility artifacts (Figures 1–3). The findings and treatment options were discussed with the patient, and due to his persistent pain and mechanical symptoms, he was scheduled for a diagnostic arthroscopy.

**Figure 4:**
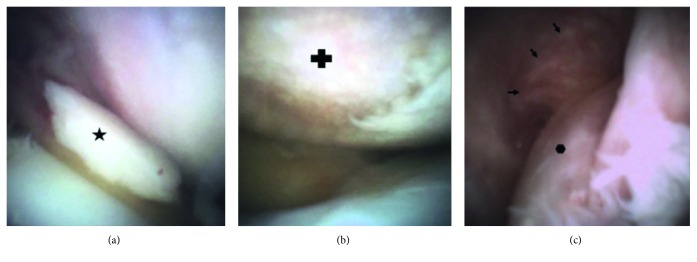
Arthroscopic images of the right knee obtained with mi-eye 2. (a) An intra-articular loose body (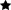) is visualized in the anterior knee. (b) A large chondral defect (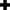) on the weight-bearing surface of the medial femoral condyle with complete loss of the articular cartilage and exposed subchondral bone. (c) A view of the intercondylar notch showing a tear of the ACL (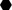) with the remnant fibers of the femoral origin (arrows) along the lateral wall of the notch.

Prior to his surgery, we were afforded the opportunity to use a new diagnostic needle arthroscopy (mi-eye 2™; Trice Medical, King of Prussia, PA) to evaluate his knee joint. After written consent was obtained, the patient's right knee was prepped in a standard sterile fashion, the subcutaneous tissue was infiltrated with 5 cc of 1% lidocaine, and mi-eye 2 was inserted into the lateral joint space. The mi-eye 2 quickly confirmed the presence of a complex, degenerative tear of the lateral meniscus. Upon further examination, we discovered an ACL tear, an intra-articular loose body, and a grade IV chondral lesion on the medial femoral condyle measuring approximately 20 mm × 20 mm (Figure 4). The mi-eye 2 provided direct visualization of the knee joint without requiring a formal operating room procedure and yielded an immediate diagnosis of the patient's intra-articular pathology, which was either equivocal or not evident on MRI.

As a result of the findings, the surgical plan was changed, and the patient subsequently underwent an arthroscopic partial lateral meniscectomy, allograft ACL reconstruction, and osteochondral allograft transplantation. The patient had an uncomplicated postoperative course and was discharged on the day of surgery. Knee ROM exercises were started immediately. He was managed with non-weight-bearing restrictions for 3 weeks, followed by progressive weight bearing from weeks 3 to 6, and then full weight bearing beginning 6 weeks after surgery.

## 3. Discussion

Knee injuries are very common with a significant percentage involving the menisci and articular cartilage. In the United States, more than 950,000 arthroscopic surgeries are performed annually on the knee alone [1]. Of these, nearly half are for medial and/or lateral meniscal injuries, with annual direct medical costs estimated at $4 billion [2, 3]. Magnetic resonance imaging is commonly used to aid in the diagnosis of internal derangement of the knee due to its superior soft tissue resolution. With reported accuracy rates of 90% or greater, the results of MRI frequently play a role in determining surgical or conservative management for patients [4, 5].

A closer review of the literature reveals a rather wide range of reported sensitivities and specificities for the diagnostic performance of MRI for intra-articular pathology of the knee [6]. The differences in reported values illustrate that MRI is not without drawbacks. Its reliability and accuracy depend on multiple factors, including equipment, sequencing protocols, radiologist's experience, and location of the pathology. One important drawback is its relatively high incidence of false negatives and false positives, which can lead to missed or faulty diagnoses and potentially result in unnecessary surgery. A study comparing the MRI and corresponding arthroscopy reports in 139 military recruits reported false positive values ranging from 65% for the medial meniscus to 42% for the articular cartilage. Furthermore, 32% of surgically treated knees were normal, despite gross pathologic findings on MRI [7].

Some studies have questioned the ability of MRI to accurately detect and characterize the size of articular cartilage defects. A study comparing the MRI reports from musculoskeletal radiologists to arthroscopic findings in 82 patients found the MRI reports missed 55% of all chondral lesions [8]. Gomoll et al. investigated the disparity between intraoperative measurements of chondral defect size and preoperative MRI size estimates in 37 patients who underwent open cartilage repair. They found that 85% of all defects were larger than predicted on MRI by an average of 65%, and only 8% of defects were accurately predicted (within 10% of final size) [9]. In a similar study of 77 patients, Campbell et al. found that 74% of defects were larger than MRI estimates, which underestimated the size by 70% on average [10]. These findings have important implications as treatment algorithms in cartilage repair are based primarily on defect size, and reliance on preoperative MRI scans alone has the potential to compromise treatment decisions. Additional considerations pertaining to the use of MRI include patients who are unable to obtain an MRI (i.e., due to a pacemaker, aneurysm clips, and severe claustrophobia) as well as the relatively high associated cost. There is undoubtedly a role for an alternative diagnostic modality, which may mitigate some of the issues mentioned above.

Small-bore (needle) arthroscopy represents an alternative diagnostic tool to assist in obtaining an accurate and timely diagnosis. Needle arthroscopy has shown to be a safe and effective means of obtaining direct visualization of a joint [11, 12]. The mi-eye 2 is an in-office diagnostic needle arthroscope consisting of a retractable 14-gauge needle, an integrated camera, and a light source, combined in a single-use device. The images are displayed on a high-definition tablet, which allows for still pictures and video recording. With this device, we were able to obtain an immediate and definitive diagnosis in a situation that would have otherwise required a formal diagnostic arthroscopy in the operating room, thus saving the patient from a general anesthesia event. The mi-eye 2 greatly expedited the time to diagnose and treat our patient's pathology. Additionally, visualizing the previously uncertain and unexpected pathology prior to the operating room allowed us to appropriately adjust the surgical plan, discuss treatment expectations and outcomes with the patient, and have the necessary instruments and implants available during surgery.

A timelier and definitive diagnosis and treatment plan, combined with fewer office visits and decreasing potentially unnecessary diagnostic studies and surgeries, can result in a significant reduction in health care costs. In fact, the use of in-office arthroscopy in place of MRI for patients presenting with meniscal pathology was reported to result in a net cost saving of $151 million annually [13]. The ability to directly visualize inside a patient's joint while they are awake provides the patient the opportunity to view and review the images in real time and be actively involved in their diagnosis and treatment. This can result in an improved patient experience and help foster a healthy relationship between the patient and surgeon.

## 4. Conclusion

We present a patient with a lateral meniscus tear, medial femoral condyle chondral defect, and ACL tear that MRI was unable to detect due to metallic artifact image distortion from a patellofemoral arthroplasty. An in-office needle arthroscopy provided a definitive diagnosis and prevented an unnecessary surgery. The in-office needle arthroscopy can be a very valuable tool in the diagnosis and treatment of intra-articular pathology, offering distinct benefits for both the orthopaedic surgeon and patient, as highlighted in our care of this patient.
